# Estimation of Landsat-like daily evapotranspiration for crop water consumption monitoring using TSEB model and data fusion

**DOI:** 10.1371/journal.pone.0267811

**Published:** 2022-05-19

**Authors:** Dong Chen, Qifeng Zhuang, Wenjie Zhang, Liang Zhu

**Affiliations:** 1 College of Geomatics Science and Technology, Nanjing Tech University, Nanjing, China; 2 Aerospace Information Research Institute, Chinese Academy of Sciences, Beijing, China; Soil and Water Resources Institute ELGO-DIMITRA, GREECE

## Abstract

Evapotranspiration (ET) plays an essential role in agricultural water resource management. Understanding regional agricultural water consumption characteristics can be improved by predicting ET using remote sensing. However, due to the lack of high-resolution images on clear-sky days or the limitation of ET reconstruction on cloudy-sky days, it remains challenging to continuously derive ET at the field scale. In this study, the Landsat and MODIS data were initially fused to obtain the Landsat-like vegetation index and land surface temperature on clear-sky days. Then the two-source energy balance (TSEB) model was applied to calculate the daily ET during the clear-sky. A canopy resistance-based gap-filling method was involved in reconstructing regional ET on cloudy days while considering different environmental factors. The estimations were validated by automatic weather system data (AWS) and eddy covariance (EC) measurements in Guantao County. The results demonstrated that the proposed scheme performed well in estimating cropland ET, with an RMSE of 0.86 mm·d^−1^ and an R^2^ of 0.65, and the NSE and PBias were 0.61 and -0.29%, respectively. The crop water consumption analysis revealed that the daily ET of winter wheat peaked during the maturation stage. Nevertheless, summer maize water consumption peaked in the middle of the growing season in this area. The temperature during the early development stage and the soil moisture in the mid and late growth stages had the greatest impact on the ET of winter wheat. During the entire growing period, soil moisture had the largest effect on the ET of summer maize. The findings showed that the TSEB model can be effectively applied to field-scale water consumption monitoring in North China through MODIS and Landsat data fusion and ET temporal reconstruction considering environmental factors.

## 1. Introduction

Evapotranspiration (ET) is an essential medium of the interaction between the surface and the atmosphere [1, 2]. It plays a crucial role in agricultural systems’ energy and water balance. Accurate estimation or measurement of ET is significant for increasing agricultural water-use efficiency and optimizing the structure of regional water use.

Reliable determination of ET is a prerequisite for realizing the effective management of water resources and rational distribution of agricultural irrigation water. At the site scale, ET can be measured by the large aperture scintillator (LAS), eddy covariance (EC), Bowen ratio, lysimeter, and pot evaporation [2]. On the other hand, ground-based measurements require many human and material resources and have restricted spatial representation. Ground-based measurements can only obtain energy fluxes over small regions and are unsuitable for large-scale research areas [3]. Remote sensing technology can provide many parameters at a regional scale, such as the vegetation index, land surface temperature, and surface albedo, which are the essential driving factors of ET estimation. Many remote sensing models have been widely validated on various land use types worldwide, showing good application prospects. The surface energy balance (SEB) model initially computed each surface energy balance component. According to the surface energy balance equation, the net radiation flux (*Rn*), soil heat flux (*G*), sensible heat flux (*H*), and latent heat flux (*LE*) constitute the surface energy balance components together [4], i.e., *LE = Rn-G-H*. The Surface Energy Balance Algorithm for Land (SEBAL) [5] and Surface Energy Balance System (SEBS) [6] are typical single-source SEB models. These models regard the surface as a large leaf and derive the sensible heat flux from the land surface radiometric temperature. The aerodynamic temperature is considered the air temperature at the source/sink level. However, theoretically, the aerodynamic temperature may significantly differ from radiation and act as a medium for transferring heat from the surface to the atmosphere [4, 7–10]. Many past studies have shown that multisource SEB models have better performance under partial vegetation cover [11, 12], e.g., the two-source energy balance model (TSEB) [13] and the Penman–Monteith (PM)-based MODIS evapotranspiration product algorithm (MOD16) [14].

However, it is still challenging to map daily ET at the field scale. The key step of this method is to solve the problem of ET reconstruction on cloudy days [15]. The reference ET (ET_ETrF) and the canopy resistance methods are standard methods for daily evapotranspiration [16–19]. The ET_ETrF approach regards the ratio (ETrF) between reference ET (ETr) and actual ET as having linear relationships between two adjacent clear days. The ETrF of cloudy days can be reconstructed by applying the linear interpolation method. The ET_ETrF method has large uncertainty because it does not consider the impact of environmental factors on cloudy days. The linear interpolation may cause significant deviations with insufficient ETrF on clear days. The canopy resistance of cloudy-sky days can be computed with the canopy resistance of adjacent clear days, leaf area index (LAI), and daily environmental factors [14, 20]. The canopy resistance-based ET temporal reconstruction method has been used for long-term crop water consumption monitoring and ET production [21].

In recent years, the North China Plain, a semiarid and semihumid environment, has been confronted with a tricky water shortage problem because crop irrigation consumes plenty of water [22–25]. Crop ET research in this area is significant for agricultural water resource management and rational utilization. In this study, the MODIS and Landsat data were initially fused to obtain the Landsat-like land surface temperature (LST) and vegetation index on clear days in this research. Then the TSEB model was used to calculate the clear days’ ET. The cloudy days’ ET was reconstructed by introducing a canopy resistance model considering the influence of daily environmental factors. The results were validated by observations from automatic weather station (AWS) and eddy covariance (EC) measurements in this study area [26, 27]. Finally, the water consumption characteristics of the main crops and the environmental factors’ influence on crop water consumption were discussed.

This study intended to generate daily Landsat-like ET using the TSEB model for crop water consumption through MODIS and Landsat data fusion in North China. Section 2 introduces the research area, remote senisng data and station measurements. Then the data fusion scheme and ET temporal reconstruction method for TSEB were proposed. In Section 3, the performance of ET estimates was systematically evaluated with surface energy observations and daily ET measurements from AWS and EC stations. The crop water consumption was analyzed according to the Landsat-like ET. Section 4 discussed the uncertainty of the TSEB model and analyzed the impact of environmental factors on crop water consumption in different growing seasons. In Section 5, we conclude the achievements for crop water consumption monitoring in Guantao County, North China.

## 2. Materials and methods

### 2.1. Study area

The North China Plain (N35.02°–N40.41°, E113.18°–E119.82°) is one of the three major plains in China, with the largest population and acts as main grain-producing region in China. Guantao County is located in the North China Plain, with a semiarid and semihumid mainland monsoon climate. The yearly mean temperature, annual potential evaporation, and precipitation are approximately 14°C, 1100.65 mm, and 548.7 mm, respectively.

**Fig 1:**
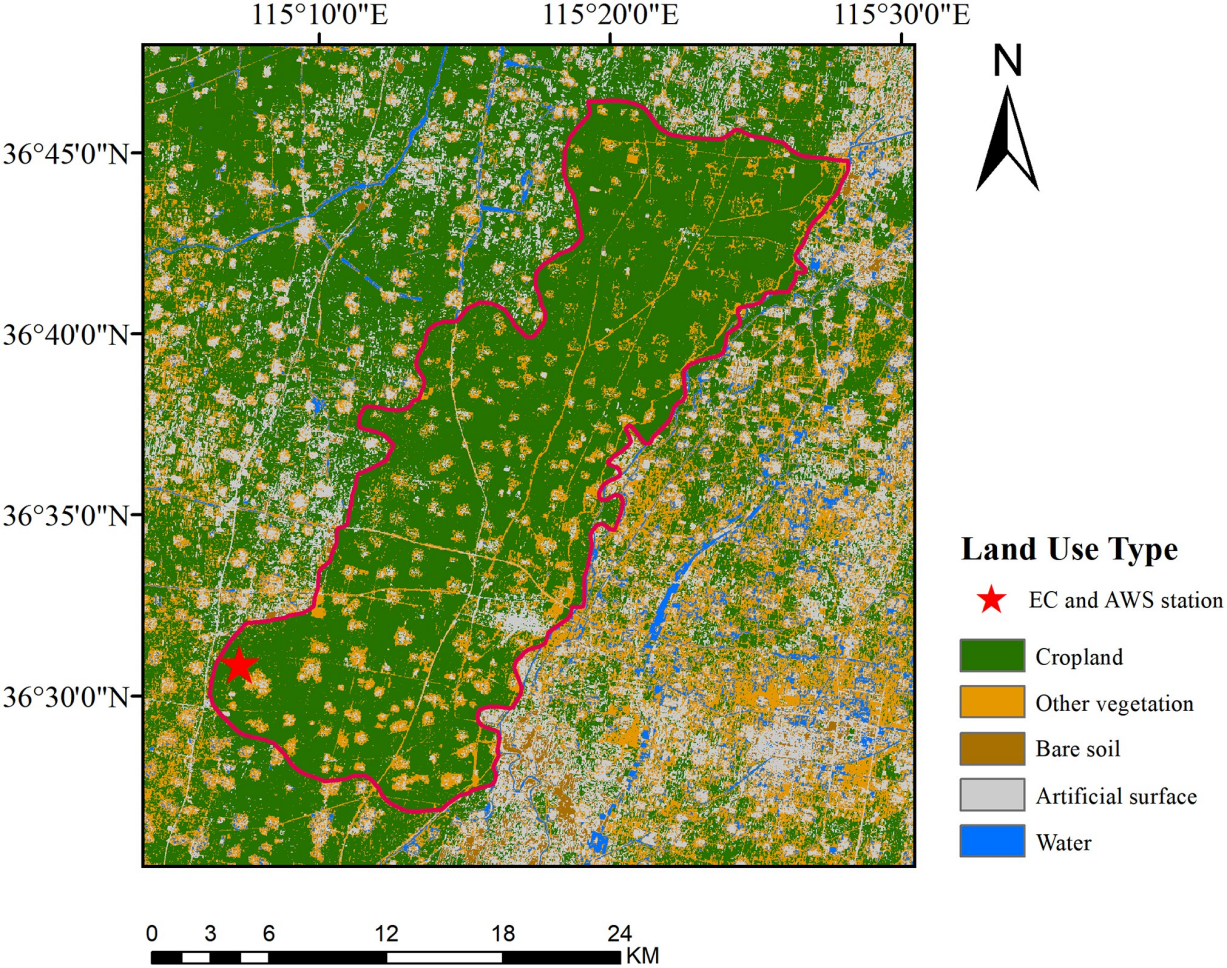
The land-use type in the Guantao area.

Due to the large amount of agricultural water consumption and insufficient precipitation, crop irrigation water is facing a significant shortage. Therefore, a large amount of groundwater is exploited in this area to supplement irrigation. The main planting form in this area is the rotation of summer maize and winter wheat. The winter wheat mainly grows from October to May, and the summer maize grows from June to September. The land-use types for the study area are shown in Fig 1, and cropland is the dominant land type in this area. The red star in Fig 1 is the automatic weather station (AWS) and eddy covariance (EC) station.

### 2.2. EC and AWS measurements

The EC and AWS stations’ longitudes and latitudes are 115.1 E, 36.5 N, respectively. The ground-based measurements from 2008 to 2010 were used to validate the results of fused Landsat-like LST and simulated daily ET [26, 27]. EdiRe software was adopted to deal with the raw EC observations, including spike detection, lag correction of H_2_O/CO^2^ relative to the vertical wind component, sonic virtual temperature correction, coordinating rotation, correction for density fluctuation, and frequency response correction. The final released EC measurements provided half-hourly sensible heat flux (*H*) and latent heat flux (*LE*).

The AWS measured the surface net radiation (Rn), wind speed, air temperature, and humidity. The soil heat flow plate was buried underground to measure the soil heat flux (*G*), mean soil temperature, and soil moisture. The surface temperature from AWS was measured by a thermal infrared thermometer. The AWS observations were averaged to half-hour frequency to be consistent with the EC data.

The Bowen ratio correction method was used to solve the energy non-closure, which assumes an invariable specific value between latent heat and sensible heat in the whole closure correction process [28]. Last, continuous daily ET measurements can be obtained by applying the mean diurnal variation (MDV) method to the EC latent heat gap-filling [29].

### 2.3. The Satellite and GLDAS data

The LST data from the MOD11A1 product at 1 km spatial resolution and land surface reflectance data from the MOD09GA at 500 m spatial resolution were applied to extract fusion parameters, i.e., the surface radiometric temperature and normalized difference vegetation index (NDVI).

The spatial resolution of Landsat 5 is 30 m in optical bands and 120 m in the thermal infrared band. The data were atmospherically corrected by the Pixel Information Expert (PIE) remote sensing image processing platform to derive the surface reflectance. The surface albedo *α* was computed according to Liang et al. [30]. The Landsat red and near-infrared bands’ reflectance was used to calculate NDVI. In this research, the surface radiometric temperature *T*_*rad*_ of Landsat 5 was computed by band six based on the single-channel algorithm put forward by Sobrino et al. [31]. The atmospheric correction parameters can be achieved on the NASA official website from the Atmospheric Correction Parameter Calculator.

The Global Land Data Assimilation System (GLDAS) contains various global-scale meteorological elements. The data used in this article included surface air pressure, specific humidity, soil moisture content (0–10 cm), downward shortwave radiation flux, air temperature, and wind velocity. We resampled all the data to a 30 m spatial resolution by bilinear interpolation. Although GLDAS has a coarse resolution, it can still provide meteorological element values of several pixels in the study area, which is more spatially representative than the information of one meteorological station.

### 2.4. Methods

**Fig 2:**
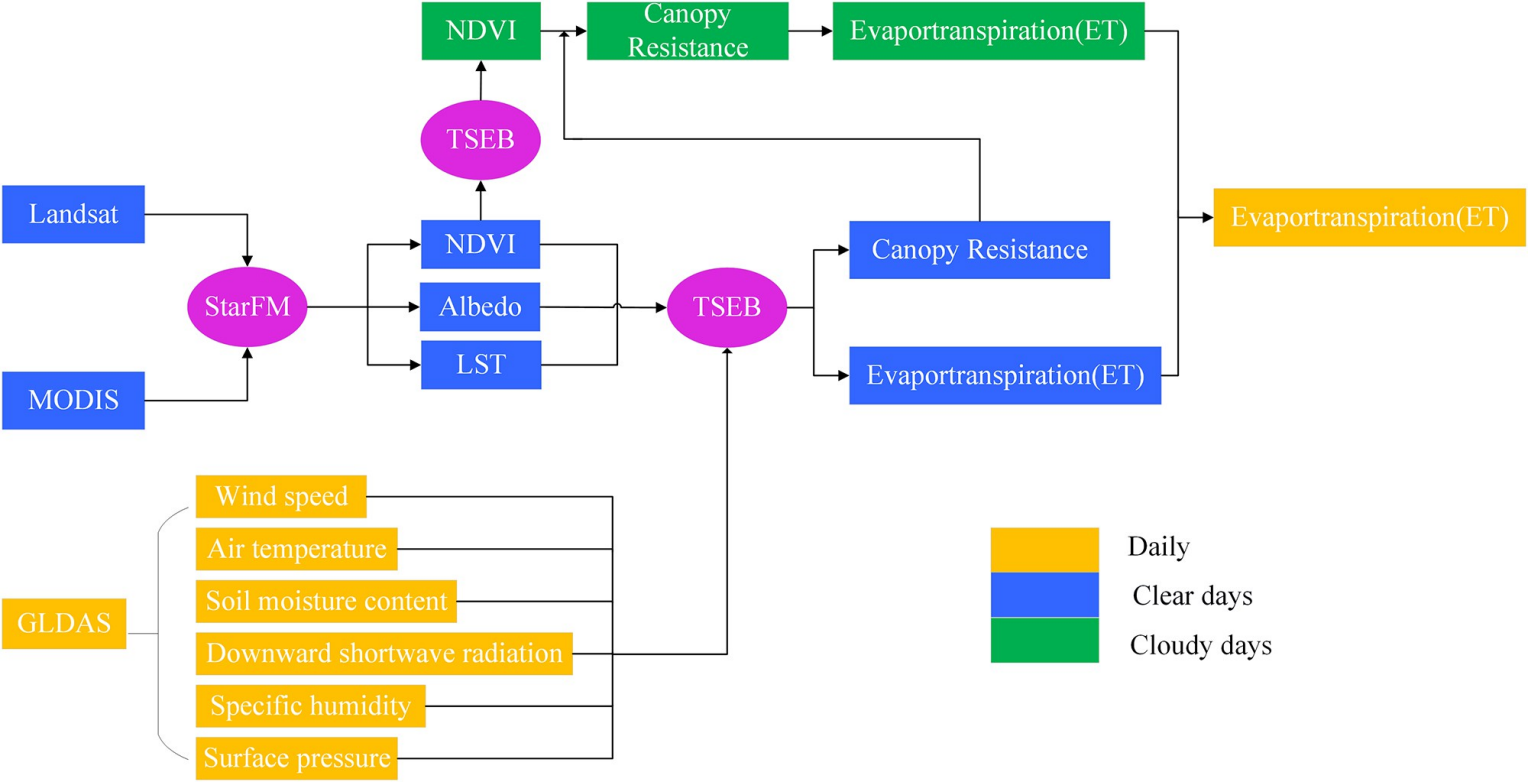
Flow chart of estimating high temporal (daily) and Landsat-like spatial resolution (30 m) ET using the TSEB model with Landsat 5 and MODIS data.

This research applied the spatial and temporal adaptive reflectance fusion model (STARFM) to obtain the NDVI and LST from clear days’ Landsat and MODIS data [32]. The acquisition of fused NDVI and LST using the same pixel searching processes. Then, the clear days’ ET and canopy resistances were calculated and inversed according to the TSEB and Penman–Monteith (PM) models, respectively [33]. The canopy resistances of the cloudy days were reconstructed to obtain continuous crop ET at the field scale by the daily environmental factors and the clear days’ canopy resistances. Fig 2 shows the overall method flow chart of this research.

#### 2.4.1 Data fusion model

The relatively high spatial resolution remote sensing image, e.g., Landsat 5, has a long revisit cycle. The cloud contamination of images resulted in no observation records available within the study area during the growing season. The MODIS satellite sensor can obtain overpass data every day, but the spatial resolution is low. Gao et al. [32] proposed STARFM to fuse surface reflectance from Landsat and MODIS. The basic idea of the model is to combine the advantages of Landsat’s high spatial resolution and MODIS’s high temporal resolution. This research fused the MODIS and Landsat NDVI and LST from 2008 to 2010 to obtain clear day data at a Landsat-like spatial resolution.

#### 2.4.2 TSEB model

The two-source energy balance (TSEB) model has been applied worldwide [34]. It uses the net radiation *Rn*, sensible heat flux *H*, and latent heat flux *LE* as energy components. The relationship between them is as follows:

$$R_{n}=R_{nc}+R_{ns}=G+H+LE$$ (1)

$$R_{nsd}=(1-\alpha )S_{d}+\varepsilon_{a}\sigma T_{a}^{4}-\varepsilon \sigma T_{rad}^{4}$$ (2)

$$R_{nc}=R_{n}[1-\text{exp}(-kLAI/\sqrt{2\;\text{cos}(\theta_{z}}))]$$ (3)

$$R_{nc}=LE_{c}+H_{c}$$ (4)

$$R_{ns}=LE_{s}+H_{s}+G$$ (5)

$$G=c_{g}R_{ns}$$ (6)

where *R*_*nc*_ and *R*_*ns*_ are the net radiation over the canopy and soil (W·m^−2^); *H*_*c*_ and *H*_*s*_ are the sensible heat flux over the canopy and soil, respectively; *LE*_*c*_ and *LE*_*s*_ are the canopy and soil radiation, respectively; and *G* is the soil heat flux. It is supposed to be an invariable ratio of *R*_*ns*_ [12], and subscripts c and s are defined as vegetation and soil, respectively. *S*_*d*_ is the downwelling shortwave radiation (W·m^−2^); *ε* is the land surface emissivity; *ε*_*a*_ is the atmospheric emissivity computed by the air temperature *T*_*a*_ (K) and water vapor pressure [35]. In Eq (3) , LAI is the leaf area index and has a linear relationship with NDVI [12]. *k* is an extinction coefficient for LAI related to the canopy structure [36]. *θ*_*z*_ is the solar zenith angle. *c*_*g*_ is assumed to be a constant ratio equal to 0.35. *H*_*c*_ and *H*_*s*_ are computed by Eqs (7) and (8), *ρ* is the air density (kg·m^−3^), *C*_*p*_ is the heat capacity of air under constant pressure(J·kg^−1^·K^−1^), and *T*_*c*_ and *T*_*s*_ are the radiometric temperatures from the canopy and the soil surface, respectively. *r*_*a*,*c*_ is the aerodynamic resistance to heat transfer between the canopy and the reference height, and *r*_*a*,*s*_ is the aerodynamic resistance over the soil surface according to Morillas et al. [37]:

$$H_{c}=\rho C_{p}\frac{T_{c}-T_{a}}{r_{a,c}}$$ (7)

$$H_{s}=\rho C_{p}\frac{T_{s}-T_{a}}{r_{a,c}+r_{a,s}}$$ (8)

$$T_{rad}={\left[f_{c}T_{c}^{4}+[1-f_{c}]T_{s}^{4}\right]}^{{}^{\frac{1}{4}}}$$ (9)

*f*_*c*_ is estimated by the fractional vegetation cover considering the sensor view angle. *T*_*c*_ and *T*_*s*_ are the canopy and soil radiometric temperatures, respectively. The TSEB uses the Priestley-Taylor (P–T) method (Equations (4) and (7)) to calculate the initial canopy temperature *T*_*ci*_:

$$T_{ci}=T_{a}+\frac{R_{nc}r_{a,c}}{\rho C_{p}}(1-\alpha_{pt}f_{g}\frac{\Delta }{\Delta +\gamma })$$ (10)

where *f*_*g*_ is the fraction of the green vegetation, *α*_*p*t_ has an initial value of 1.26. *γ* is the psychrometric constant of ~0.066 (kpa·K^−1^), and Δ is the slope of the saturation vapor pressure versus the air temperature curve. *H*_*c*_ and *H*_*s*_ can be calculated by combining Eqs (7) and (8) and (9) with the initial *T*_*c*_, and *LE*_*s*_ are computed by Eqs (1)–(5). If the value of *LE*_*s*_ is negative, an iterative process is adopted to produce a new estimate of *T*_*c*_ by the decline of *α*_*p*t_.

The evaporative fraction (EF) method was used to extend the instantaneous evapotranspiration to the daily scale. It assumes that the evaporative fraction, i.e., *LE*/(*Rn*-*G*) is a constant value during the daytime. *L* is the latent heat of vaporization (MJ·m^-2^·mm^-1^). The daily net radiation (*R*_*n24*_, MJ·m^-2^·d^-1^) was computed by the solar radiation from the GLDAS data.

$$ET_{24}=EF\frac{R_{n24}}{L}=\frac{LE}{R_{n}-G}\times \frac{R_{n24}}{L}$$ (11)

$$LE_{24}=\frac{(R_{n24}-G_{24})\times LE}{R_{n}-G}$$ (12)

*G*_*24*_ is defined as the daily average soil heat flux and is approximately equal to zero. *LE*_*24*_ is the daily latent heat flux.

$$R_{cunc}=\frac{LAI_{clr}\times R_{cclr}}{LAI_{unc}\times m(T\;\text{min})\times m(VPD)}$$ (13)

*R*_*cclr*_ and *R*_*cunc*_ are the clear days’ and cloudy days’ canopy resistance, respectively. The former is calculated by inversing the TSEB clear days’ ET into the Penman–Monteith equation. This study used Savitzky–Golay filtering to smooth the fused Landsat-like NDVI. Then the daily NDVI data were used to calculate the daily LAI. *m(Tmin)* and *m(VPD)* are two restriction functions used to express extreme temperature and humidity conditions on the opening and closing of plant stomata [38]. *m(Tmin)* and *m(VPD)* were used to limit potential stomatal conductance by minimum air temperatures (Tmin) and reduce the potential stomatal conductance when VPD is high enough to inhibit photosynthesis, respectively. Therefore, by introducing the canopy resistances of cloudy days into the TSEB model, the ET on cloudy days can be obtained. Table 1 summarizes the parameters used in this study.

**Table 1:** Parameters used in this study.

Parameter	Definition	Parameter	Definition
**ET**	Evapotranspiration	***θ***_***z***_	Solar Zenith Angle
**Rn**	Net Radiation	***ρ***	Air Density
**G**	Soil Heat Flux	***C***_***p***_	Heat Capacity of Air under Constant Pressure
**H**	Sensible Heat Flux	***T***_*a*_	Air temperature
**LE**	Latent Heat Flux	***T***_***c***_	Radiometric Temperatures from the Canopy
**LST**	Land Surface Temperature	***T***_***s***_	Radiometric Temperatures from the Soil Surface
**LAI**	Leaf Area Index	***r***_***a*,*c***_	Aerodynamic Resistance to Heat Transfer between the Canopy and the Reference Height
**NDVI**	Normalized Difference Vegetation Index	***r***_***a*,*s***_	Aerodynamic Resistance over the Soil Surface
***S***_***d***_	Downwelling Shortwave Radiation	***f***_***g***_	Fraction of the Green Vegetation
***ε***	Land Surface Emissivity	***γ***	Psychrometric Constant
***ε***_***a***_	Atmosphere Emissivity	**Δ**	Slope of the Saturation Vapor Pressure Versus the Air Temperature Curve

## 3. Results

### 3.1. Landsat-like fused-LST

**Fig 3:**
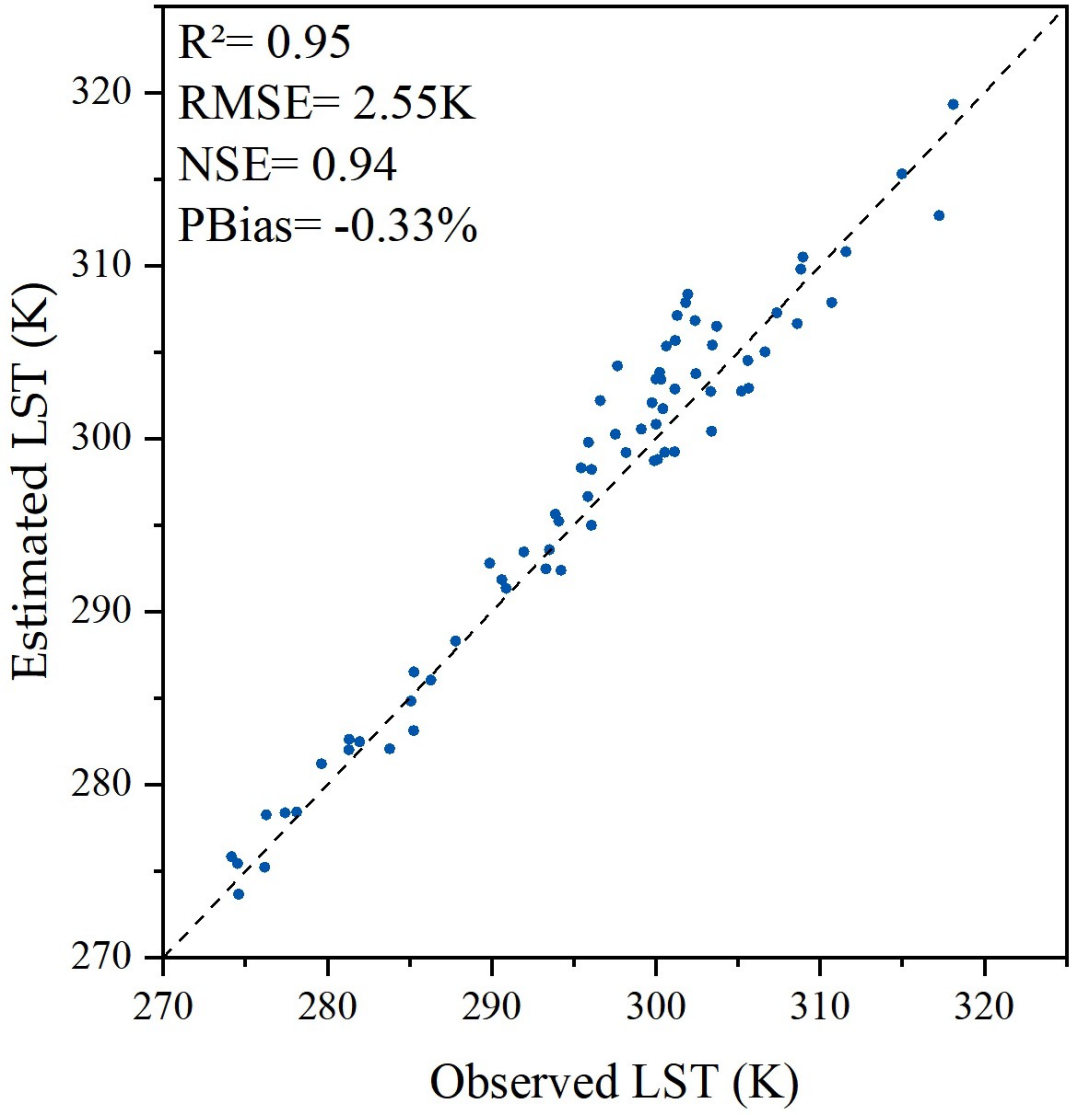
Validation of LST from STARFM simulation and LST from AWS observations.

The LST fused by the STARFM model was compared with the ground observations. The determination coefficient (R^2^) between fused-LST and the surface temperatures measured by AWS shown in Fig 3 was 0.95. The percentage bias (PBias) and the root mean square error (RMSE) were −0.33% and 2.55 K, respectively. The result indicated that although fused-LST cannot entirely reflect the actual temperature of the surface, the fused-LST obtained by STARFM has reasonable accuracy.

### 3.2. Daily net radiation

**Fig 4:**
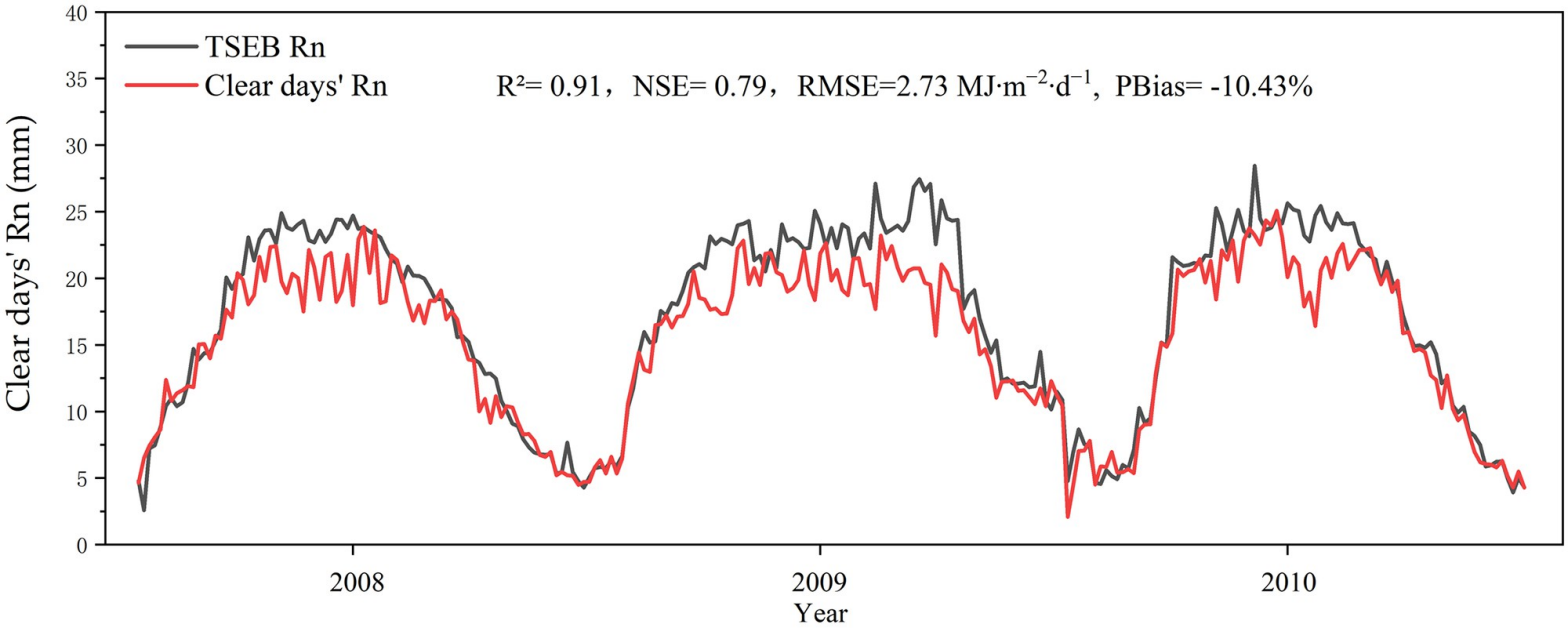
Validation of daily Rn from the TSEB model and daily Rn from AWS observations.

The daily net radiation flux has a considerable influence on the estimation of daily ET. The TSEB model used daily net radiation (Rn) as a primary boundary condition. The daily net radiation computed from the GLDAS solar radiation and surface albedo was compared with the AWS measurements. Fig 4 shows the overall Nash–Sutcliffe efficiency (NSE), root mean square error (RMSE), coefficient of determination (R^2^), and percent bias (PBias) statistics for daily net radiation estimations. The R^2^, NSE, RMSE, and PBias were 0.91, 0.79, and 2.73 MJ∙m^−2^∙d^−1^, and -10.43%. An NSE value close to 1 represents the overall high simulation accuracy of the model. The negative PBias values indicated that the TSEB model overestimated the daily net radiation. Similar to Santhi et al. (2001) [39], the accuracy was regarded as reasonable if the NSE and R^2^ were more significant than 0.5 and 0.6, respectively.

### 3.3. Validation of daily ET

**Fig 5:**
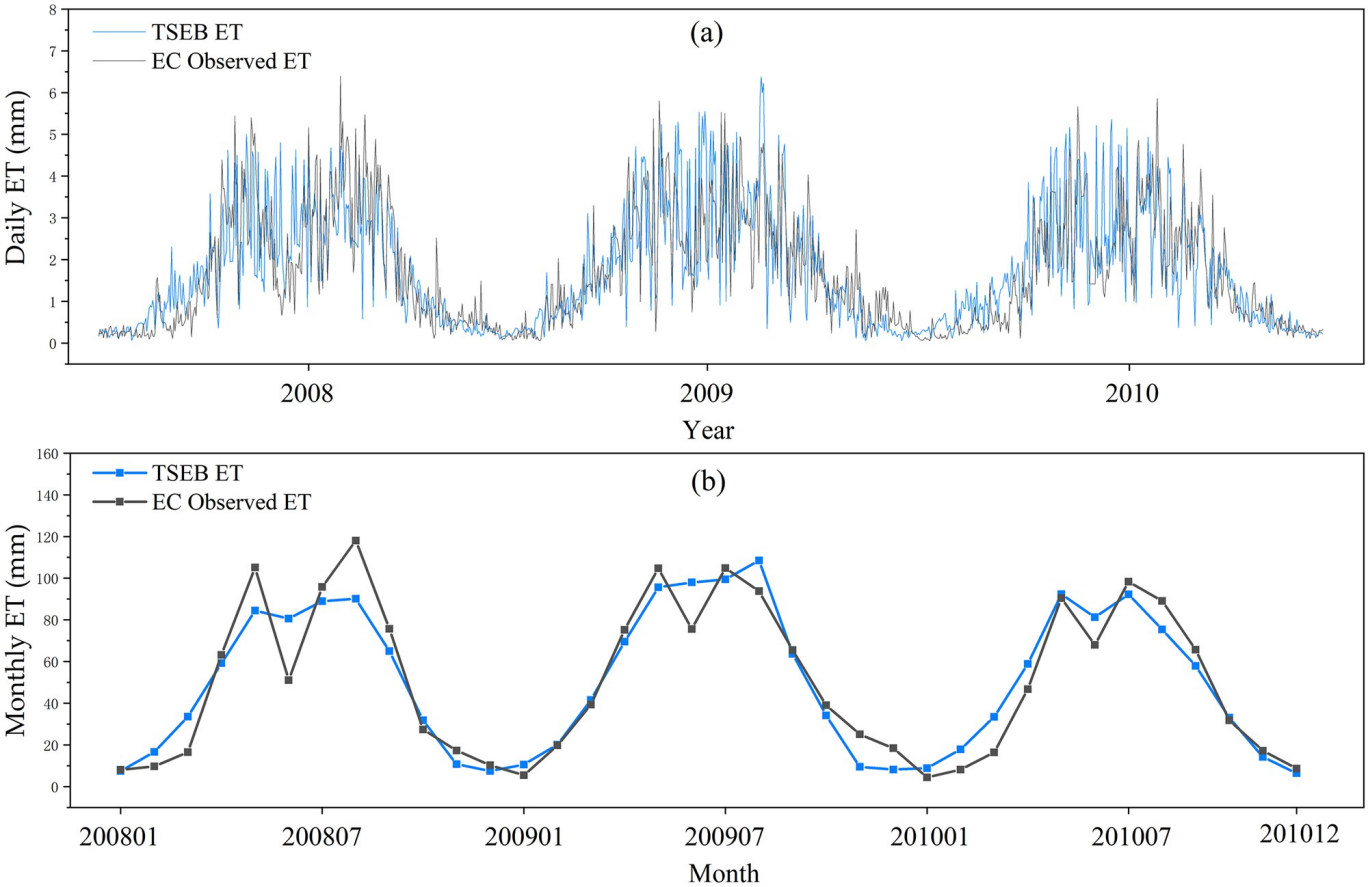
Comparative analysis of simulated daily ET (a) and monthly ET (b) with EC measured ground truth.

The daily ET computed from TSEB was compared with the EC measurements from 2008 to 2010. As shown in Fig 5(a) and Table 2, the TSEB shows a good performance of daily ET compared to the land truth from EC observations. As a whole, the R², NSE, and RMSE of the estimated daily ET at the field scale were 0.65, 0.61, and 0.86 mm, respectively, and had no noticeable interannual differences from 2008 to 2010. The overall PBias was 0.29%, and the results in 2009 had the best PBias (0.5%).

**Table 2:** The general performance results of simulated daily evapotranspiration from 2008 to 2010.

Method	Temporal Scale	Years	NSE	R^2^	RMSE	PBias
					(mm)	(%)
TSEB	Daily	2008	0.65	0.65	0.87	3.64
		2009	0.57	0.67	0.87	0.5
		2010	0.6	0.64	0.84	-5.58
		Overall	0.61	0.65	0.86	-0.29
	Monthly		0.89	0.89	11.71	-0.27

The change characteristics of monthly ET obtained by TSEB and EC observations are demonstrated in Fig 5(b). The values of the NSE, R^*2*^, and PBias on the monthly scale became larger than those on the daily scale. These validation results showed that using the proposed method in this research can effectively estimate continuous ET at the field scale.

### 3.4. Crop water consumption

**Fig 6:**
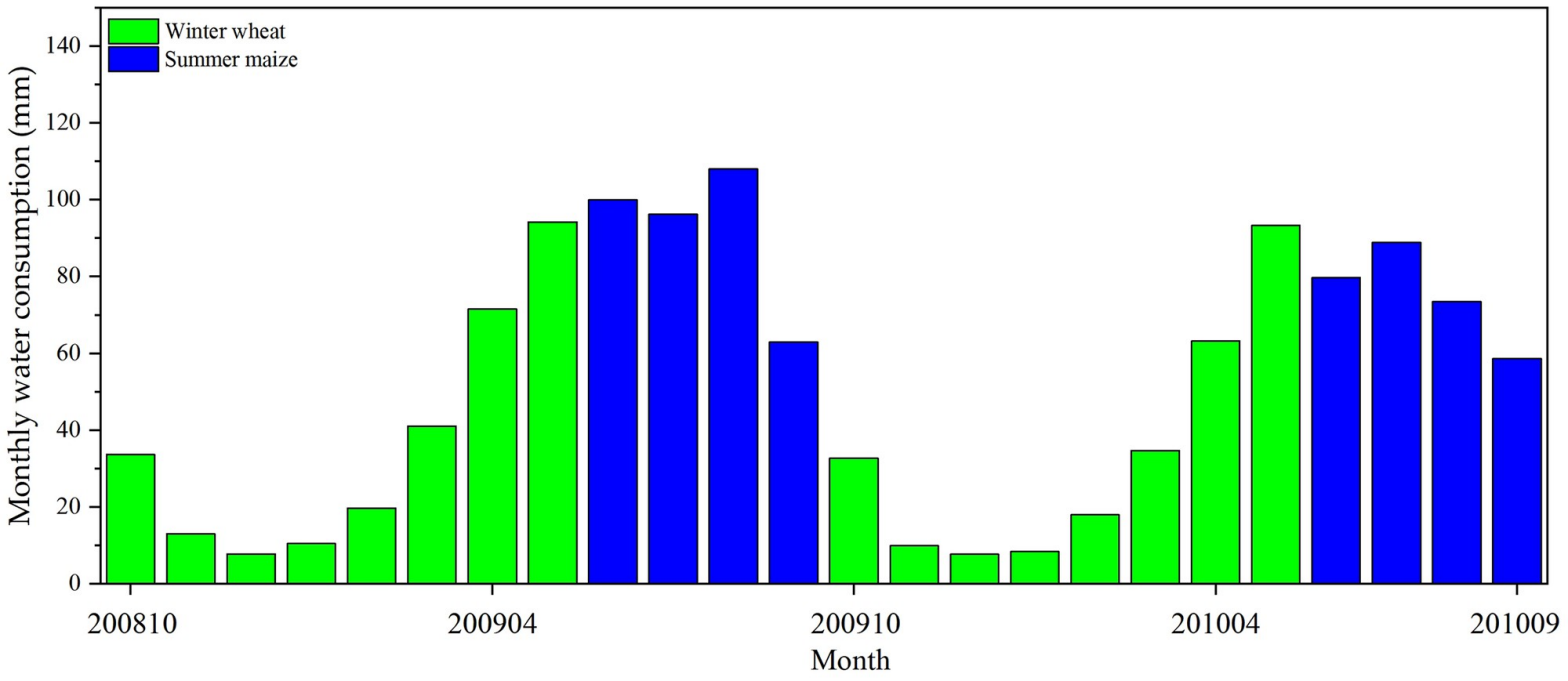
Monthly water consumption for winter wheat and summer maize.

As demonstrated in Fig 6, the water consumption of summer maize and winter wheat during the whole growth period showed noticeable monthly changes. The middle growth period of summer maize was from July to August and was the most vigorous stage. The transpiration of leaves reached a maximum during this period. However, the maturation growth stage of winter wheat in June had the highest ET. Fig 6 shows that in the early period of growth, the average water consumption of summer maize was greater than that of winter wheat. Nevertheless, the average water consumption of summer maize in the middle and late growth stages was less than that of winter wheat.

Table 3 shows the water consumption of summer maize and winter wheat at different growth stages.

**Table 3:** The general performance results of simulated daily evapotranspiration.

Crop Type	Growing Stages	Months	Water Consumption (mm)	
			2008–2009	2009–2010
**Winter Wheat**	Sowing	Oct	33.69	32.71
	Wintering	Nov to Jan	50.90	43.95
	Reviving	Feb to Mar	41.08	34.70
	Flowering	Apr	71.48	63.16
	Maturation	May	94.21	93.33
	Total	Oct to May	291.36	267.85
**Summer Maize**	Sowing	Jun	99.95	79.67
	Seeding	Jul	96.22	88.88
	Flowering	Aug	107.96	73.45
	Maturation	Sep	62.91	58.67
	Total	Jun to Sep	367.04	300.67

Table 3 shows that the total water consumption of winter wheat was less than that of summer maize throughout the growth stages. Winter wheat had similar water consumption in different growth stages from 2008 to 2009 and 2009 to 2010. The total water consumption for the two periods was 267.85 mm and 291.36 mm, respectively. However, the water consumption of summer maize varied significantly in different growth stages in 2009 and 2010. The water consumption of summer maize in 2009 was substantially greater than that in 2010; with a total water consumption for the two periods of 367.04 mm and 300.67 mm, respectively. This increase in consumption is generally due to greater evapotranspiration caused by more irrigation of cropland.

**Fig 7:**
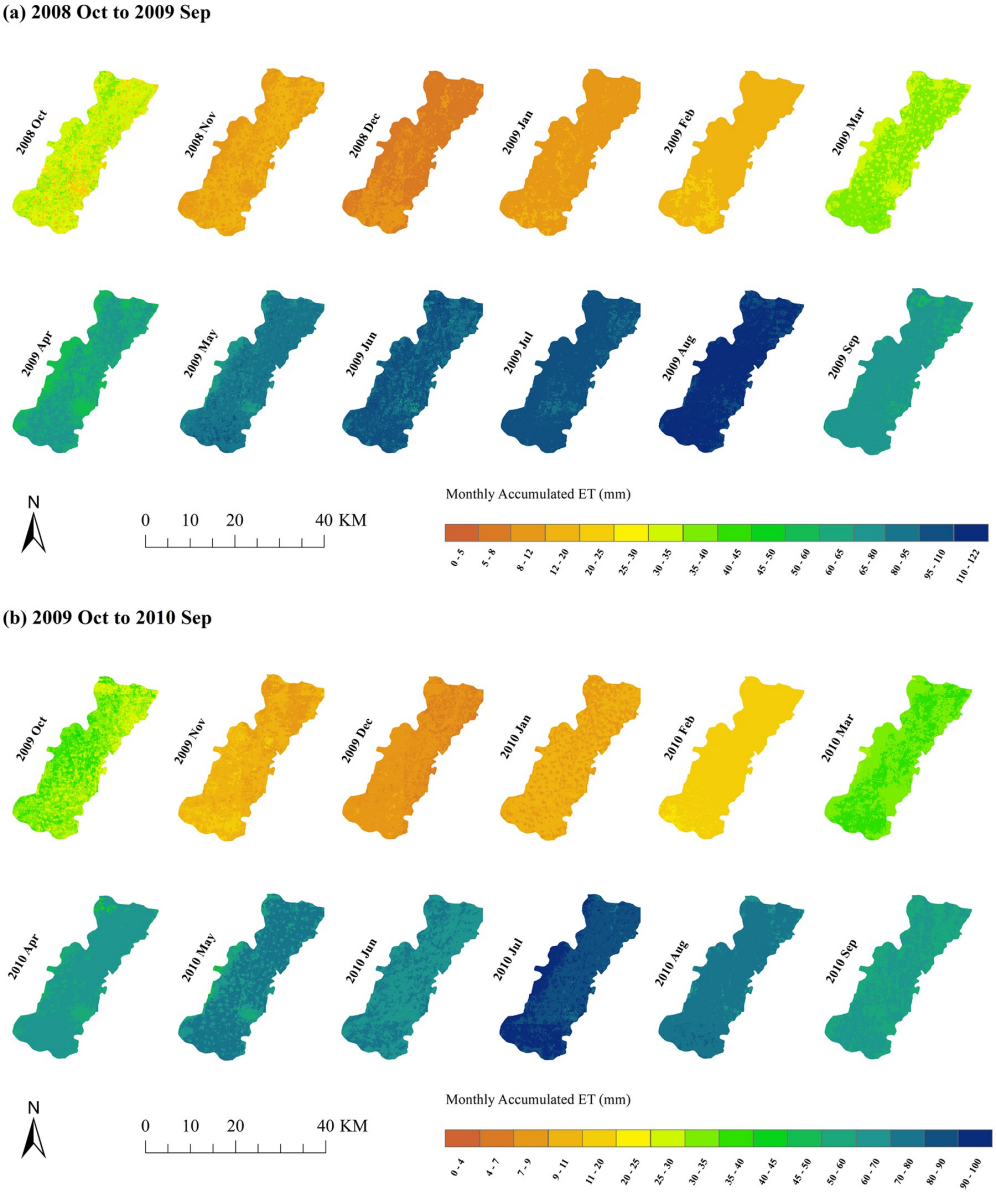
Spatial ET distribution of summer maize and winter wheat.

Fig 7 shows the cropland spatial ET distribution from 2008 Oct. to 2010 Sep. in the study area. Figs 6 and 7 show that the water consumption characteristics of the summer maize and winter wheat showed regular changes according to the month. May to August was the peak of crop water consumption, with an average of approximately 80–100 mm per month from 2008 to 2010; November to March was the trough of crop water consumption, with approximately 10 mm per month. Combined with Table 3, Fig 7 shows that the accumulated ET of summer maize had apparent spatial variations in 2009 and 2010. The accumulated ET in August 2009 was much higher than that in August 2010. The reason might be that irrigation or rainfall increases the soil moisture, and soil evaporation and vegetation transpiration become more significant. The difference in ET between irrigated and nonirrigated croplands will increase. Overall, the water consumption of summer maize is greater than that of winter wheat. In the North China Plain, rainfall is mainly concentrated during summer, and the winter wheat is more dependent on irrigation. There were also differences in water consumption between different years for both winter wheat and summer maize, which may be related to the crop planting density or irrigation method.

## 4. Discussion

High-resolution ET data can provide more details for the water consumption of plants. The application of the TSEB model in field-scale crop water consumption monitoring in North China still needs careful investigation. This study used data fusion and the TSEB model, which considers environmental factors, to estimate daily field-scale ET in the North China Plain.

The LST plays a significant role in ET estimation, but the data fusion model has uncertainty. The validation result in Fig 3 shows that the fused-LST obtained in this paper has reasonable accuracy. This is the premise that the subsequent model can perform well. As shown in Fig 4, the daily net radiation is overestimated. The reason for the overestimation of daily net radiation is generally the uncertainty of meteorological data. According to the TSEB model, the daily net radiation is inversely proportional to albedo. Although there may be a gap between meteorological data and actual values, from the results, the accuracy of daily net radiation is still within an acceptable range. The overestimation of daily net radiation also leads to the overestimation of overall ET in Table 2. The overall validation shows that the accuracy of the monthly statistical results is significantly higher than that of the daily statistical results, which explains why the estimation error of the scheme proposed by the article tends to compensate over time. This is mainly due to the random errors associated with daily evapotranspiration cancels each other out after time accumulation. Compared with previous ET research, which can only be carried out on days with clear-sky, the temporal reconstruction method proposed in this paper improved the scenario in which effective data cannot be obtained on cloudy-sky days. Through the data fusion model, the LST on cloudy-sky days was obtained, and then the ET on cloudy-sky days was estimated. When the effective LST cannot be obtained by using the data fusion model, the ET on cloudy-sky days was obtained by introducing the canopy resistance on cloudy-sky days into the TSEB model. This greatly improved the temporal resolution of ET research. The TSEB model that considers environmental factors can better analyze the variation law of the main environmental factors affecting evapotranspiration in the crop growth stages.

The results indicated that the proposed scheme could conduct accurate ET estimation at a high spatiotemporal resolution at the field scale. However, reconstruction of canopy resistance on cloudy days using clear days calculation may affect the results because the growth of crops does not fit this general linear relationship. A time interval of at least five days is indispensable for accurate daily ET reconstruction, according to Alfieri et al. (2017). Obviously, errors may occur if the gap size is more extensive [40]. Irrigation also affects evapotranspiration, increasing the irrigation amount, soil water content, and evapotranspiration. Many previous studies have shown that ET is connected to crop species and may be influenced by environmental factors. These environmental factors are complex, and the common ones are solar radiation, air humidity, air temperature, and soil moisture [10, 41].

The TSEB model includes a variety of exchange patterns between canopy and soil latent heat flux due to vegetation stress rather than directly analyzing the impact of environmental factors. The feedback of the TSEB model has a difference in environmental stress in semihumid and semiarid regions. Jarvis’s type equations [42, 43] were chosen to evaluate the model sensitivity and analyze the influence of environmental factors on crop growth. It contains four environmental factors, i.e., solar radiation (*f*_***sr***_) soil moisture (*f*_***sm***_), air humidity (*f*_***hu***_), and air temperature (*f*_***ta***_). The *f*_***sr***_ factor describes the influence of solar radiation and implies the energy stress for heat flux [43]. It is calculated from downward shortwave radiation. The *f*_***sm***_ factor considers the effect of soil moisture on canopy transpiration. The factor *f*_***hu***_ is related to water stress conditions and addresses the impact of vapor pressure difference (VPD) in the atmosphere. The *f*_***ta***_ factor represents the temperature constraint on canopy resistance. It describes the correlation between the air temperature at the reference height level over the canopy and the optimal air temperature (e.g., 298 K) for crop growth. Theoretically, the values of all environmental factors are between 0–1.

The mean environmental factor values for the summer maize and winter wheat throughout the entire growth stage are shown in Table 4. Winter wheat and summer maize’s corresponding mean environmental factors such as *f*_*sm*_, *f*_*hu*_, and *f*_*ta*_ differed significantly. This result indicated that the winter wheat was more susceptible to *f*_*sm*_, *f*_*hu*_, and *f*_*ta*_ when the growing environment was drier and colder than that of summer maize. In contrast, the *f*_*sr*_ for winter wheat and summer maize did not significantly differ. Soil moisture was the primary influencing factor for summer maize and winter wheat throughout the whole growing season.

**Table 4:** Mean environmental factor values for summer maize and winter wheat.

Crop Kind	Mean Environment Factors Values			
	*f*_*sm*_	*f*_*hu*_	*f*_*ta*_	*f*_*sr*_
**Winter wheat**	0.47	0.70	0.75	0.84
**Summer maize**	0.58	0.78	0.94	0.85

Tables 5 and 6 show the values of the environmental factors for winter wheat and summer maize during the three growing periods. The *f*_*sm*_ and *f*_*hu*_ had similar matters in the early growth stage of summer maize (sowing stage). Summer maize was mainly affected by soil moisture and air humidity, most likely due to irrigation; this suggested that both factors affect water vapor transfer from the canopy to the atmosphere. The air temperature was appropriate for the middle period of the summer maize growth (seeding and flowering stages). Solar radiation gradually replaced air humidity as the main factor affecting summer maize, and only soil moisture became a significant influencing factor during the maturation stage of growth. Table 6 shows that in the early period of winter wheat (sowing stage), the air temperature became the major influencing factor in this period. During the middle growth period for the winter wheat (wintering, reviving, and flowering stages), the impact of air temperature decreased because the weather warmed. During the maturation growth of winter wheat, soil moisture and air humidity were identical, which jointly became the main influencing factor for this period. The solar radiation factor was relatively high throughout the summer maize and winter wheat, indicating that solar radiation energy had a weak effect on crop evapotranspiration. Although solar radiation played a key role, the soil moisture data were not introduced to estimate the cropland evaporation. The spatial resolution of remote sensing-based soil moisture products does not meet the requirements of estimated evapotranspiration.

**Table 5:** Environmental factor values for summer maize during different periods.

Crop Kind	Growth Stage	Environment Factors			
		*f*_*sm*_	*f*_*hu*_	*f*_*ta*_	*f*_*sr*_
**Summer maize**	**Early**	0.62	0.70	0.98	0.95
	**Middle**	0.57	0.85	0.95	0.75
	**Maturation**	0.54	0.78	0.89	0.85

**Table 6:** Environmental factor values for winter wheat during different periods.

Crop Kind	Growth Stage	Environment Factors			
		*f*_*sm*_	*f*_*hu*_	*f*_*ta*_	*f*_*sr*_
**Winter wheat**	**Early**	0.43	0.82	0.57	0.93
	**Middle**	0.46	0.77	0.69	0.73
	**Maturation**	0.50	0.51	0.97	0.86

## 5. Conclusions

Accurate and continuous estimation of cropland ET is essential for agricultural irrigation scheduling, a necessary measure for the rational utilization of water resources, monitoring droughts, and aiding in scarce water supply management. It is critical in the arid and semiarid areas of North China, where water resources are limited.

This study generated Landsat-like daily ET using data fusion combined with the TSEB model for crop water consumption in Guantao County, North China. Through the fusion of MODIS and Landsat data, the high-resolution surface temperature and vegetation index data on clear days were obtained, and then the ET on cloudy days was obtained using the ET reconstruction method considering environmental factors. The validation demonstrated that this ET computation scheme performed well in crop water consumption monitoring. The overall R² is 0.65, NSE is 0.61, RMSE is 0.86 mm·d^−1^, and PBias is -0.29%. The analysis of crop water consumption showed that the water consumption of winter wheat and summer maize peaked during the mature stage and the middle of the growing season, respectively. Among them, for winter wheat, the largest factor affecting ET in the early development stage was temperature, while in the middle and late growth stages, soil moisture had the greatest impact on ET. For summer maize, soil moisture played a decisive role in the change in ET throughout the growth period.

This study demonstrates the feasibility of the TSEB combined with a data fusion model to generate daily ET for crop water consumption monitoring with a high spatial resolution (30 m). This will be a good and feasible practice for the rational allocation of water resources in the North China Plain and other similar agricultural areas globally.
